# Bee Venom Mitigates Cisplatin-Induced Nephrotoxicity by Regulating CD4^+^CD25^+^Foxp3^+^ Regulatory T Cells in Mice

**DOI:** 10.1155/2013/879845

**Published:** 2013-02-14

**Authors:** Hyunseong Kim, Gihyun Lee, Soojin Park, Hwan-Suck Chung, Hyojung Lee, Jong-Yoon Kim, Sangsoo Nam, Sun Kwang Kim, Hyunsu Bae

**Affiliations:** ^1^Department of Physiology, College of Oriental Medicine, Kyung Hee University, 1 Hoeki-Dong, Dongdaemun-gu, Seoul 130-701, Republic of Korea; ^2^Department of Internal Medicine, College of Oriental Medicine, Kyung Hee University, Seoul 130-701, Republic of Korea; ^3^Institute of Oriental Medicine, Kyung Hee University, 1 Hoeki-Dong, Dongdaemun-gu, Seoul 130-701, Republic of Korea

## Abstract

Cisplatin is used as a potent anticancer drug, but it often causes nephrotoxicity. Bee venom (BV) has been used for the treatment of various inflammatory diseases, and its renoprotective action was shown in NZB/W mice. However, little is known about whether BV has beneficial effects on cisplatin-induced nephrotoxicity and how such effects might be mediated. In the present study, the BV-injected group showed a significant increase in the population of Tregs in spleen. Although there was no significant difference in the numbers of Tregs 3 days after cisplatin injection between the BV- and PBS-injected groups, more migration of Tregs into the kidney was observed 6 hours after cisplatin administration in BV group than in PBS group. In addition, BV-injected mice showed reduced levels of serum creatinine, blood urea nitrogen, renal tissue damage, proinflammatory cytokines, and macrophage infiltration into the kidney 3 days after cisplatin administration. These renoprotective effects were abolished by the depletion of Tregs. The anticancer effect of repeated administrations of cisplatin was not affected by BV injection. These results suggest that BV has protective effects on cisplatin-induced nephrotoxicity in mice, at least in part, through the regulation of Tregs without a big influence on the antitumor effects of cisplatin.

## 1. Introduction

cis-Diamminedichloroplatinum (cisplatin) is widely used as a chemotherapeutic agent to treat various cancers [1]. It is effective against cancer of the lung, head and neck, testis, ovary, cervix, endometrium, bladder, and oropharynx [2]. However, side effects in normal tissues and organs, particularly nephrotoxicity, limit the use of cisplatin and related platinum-based therapeutics [1]. The nephrotoxic effects of cisplatin are manifested as a decrease in creatinine clearance and electrolyte imbalances, particularly hypomagnesemia, mainly due to the acute cytotoxic effects of cisplatin on proximal and distal tubules [3].

Foxp3 is an important regulator of the activation and function of CD4^+^CD25^+^ regulatory T cells (Tregs) [4]. Tregs play a pivotal role in the maintenance of tolerance in the immune system [5–7]. Recently, we provided clear evidence that Tregs mitigated cisplatin-induced nephrotoxicity. The adoptive transfer of Tregs into mice successfully protected cisplatin-induced renal damage, whereas the depletion of Tregs accelerated cisplatin toxicity [8]. From these findings, it is expected that agents capable of enhancing Treg function would have beneficial effects on cisplatin-induced nephrotoxicity. Thus, we screened a natural product library using a Foxp3 promoter reporter assay system and found that bee venom ((BV), from *Apis melifera*) significantly increased Tregs, compared to other candidates.

BV has been used for the treatment of various inflammatory diseases, such as rheumatoid arthritis, and possesses strong immune modulatory effects [9, 10]. BV injection produced a marked suppression of leukocyte migration and a significant reduction in the concentration of tumor necrosis factor (TNF) in mice [11]. Our recent study demonstrated that BV causes immune tolerance by increasing the population of Tregs in an autoimmune disease model, lupus-prone NZB/W mice [12]. However, it remains unknown whether BV mitigates cisplatin-induced nephrotoxicity in mice by regulating Tregs. 

In the present study, we examined the protective effects of BV pretreatment on renal tissue/function and Tregs in cisplatin-injected mice. We found that BV inhibited the levels of proinflammatory cytokines, renal dysfunction, and tissue damage in cisplatin-injected mice, which were due to the regulation of Tregs in the kidney. Thus, BV might have a therapeutic potential in preventing cisplatin-induced nephrotoxicity.

## 2. Materials and Methods

### 2.1. Animals

Male C57BL/6 mice (5-6 weeks old, Korea Biolink, Chungbuk, Korea), weighing 18–20 g each, were used in most of the experiments. To analyze CD4^+^CD25^+^ Foxp3^+^ Tregs in spleen and kidney, male Foxp3^EGFP^C57BL/6 mice (C. Cg-Foxp3^tm2Tch^/J, 5-6 weeks old) were purchased from The Jackson Laboratory (Bar Harbor, ME, USA). They were kept under specific pathogen-free conditions with air conditioning and a 12 h light/dark cycle. Mice had free access to food and water during experiments. This study was approved by the Animal Care and Use Committee of Kyung Hee University (KHUASP (SE)-09-009).

### 2.2. BV and Cisplatin Administration

Mice received BV at a concentration of 1 mg/kg body weight once a day for 5 days. The control group received an equal volume of PBS. Cisplatin (cis-diammineplatinum II dichloride; Sigma-Aldrich, St. Louis, MO, USA) was dissolved in 0.9% of PBS at a concentration of 1 mg/mL. Two days after the fifth administration of BV or PBS, all mice received a single intraperitoneal (i.p.) injection of cisplatin (25 mg/kg body weight). Mice were sacrificed under ether anesthesia 72 h after cisplatin administration. Blood, spleen, and kidney samples were extracted for analysis.

### 2.3. The Levels of CD4^+^CD25^+^Foxp3^+^ Tregs in Splenocytes

Splenocytes were isolated from Foxp3^EGFP^ mice for immunofluorescence and T cells were incubated with anti-mouse CD3 and anti-CD28 antibodies. The cells were treated with PBS or BV (1 *μ*g/mL) and cultured in complete RPMI 1640 media containing 2.5 *μ*g/mL anti-mouse CD3 antibody and 2 *μ*g/mL of anti-mouse CD28 antibody for 72 hr. Single-cell suspensions were incubated with fluorescently tagged Abs directed against a panel of cell surface markers and stained with CD4 and CD25 (eBioscience, San Diego, CA, USA). Foxp3^EGFP^ mice were also injected BV (1 mg/kg) or PBS once a day for 5 days and sacrificed to measure the level of Treg in splenocytes. The staining was performed in accordance with the manufacturer's instructions. All FACS data were acquired on a FACSCalibur flow cytometer (BD Biosciences, San Jose, CA, USA) and analyzed using CellQuest Pro (BD Biosciences, San Jose, CA, USA).

### 2.4. Survival Test

After cisplatin injection, mice were monitored every 6 h for 10 days. The results were statistically analyzed.

### 2.5. Assessment of Renal Dysfunctions

Blood samples were obtained at 24, 48, and 72 h after cisplatin injection. Samples were left at room temperature for 1 hour and then centrifuged for 10 min at 1000 g to obtain the serum. Renal function was assessed by measuring blood urea nitrogen (BUN) and creatinine using a FUJI DRI-CHEM 3500i instrument (Fuji Photo Film Ltd., Tokyo, Japan). 

Kidney tissues were fixed in 4% paraformaldehyde (PFA) for 1 day and then embedded in paraffin, sliced into 5 *μ*m sections and stained with hematoxylin and eosin (H&E). Three pathologists who were blinded to the experiments scored the degree of tubular injury. Renal tubular injury was assessed using a semiquantitative score in which the percentage of cortical tubules showing epithelial necrosis was assigned a score of 0, none; 1, <10%; 2, 10–25%; 3, 25–75%; or 4, >75% [13].

### 2.6. The Depletion of CD4^+^CD25^+^Foxp3^+^ Regulatory T Cells in Mice

Anti-mouse CD25 rat IgG1 (anti-CD25; clone PC61) antibodies were generated in-house from hybridomas obtained from ATCC (Manassas, VA, USA). A dose of 0.1 mg of anti-CD25 antibody was injected each day before BV and cisplatin administration. The efficacy of CD4^+^CD25^+^Foxp3^+^ Treg depletion was confirmed by flow cytometry analysis using PE-anti-mouse CD25 and FITC-anti-mouse CD4.

### 2.7. Assessment of Proinflammatory Cytokines

To examine proinflammatory cytokine levels after cisplatin administration, the TNF-*α* and IL-6 protein levels in the kidney were measured using an enzyme-linked immunosorbent assay (ELISA; BD Biosciences, San Jose, CA, USA). Frozen kidney tissue was homogenized in a buffer containing 10 mM HEPES, 10 mM KCl, 0.1 mM EGTA, 1 mM dithiothreitol, and 10 mM phenylmethanesulfonyl fluoride [14], incubated for 20 min on ice and then centrifuged at 13000 rpm (4°C) for 15 min. The supernatant was evaluated using a kidney inflammatory cytokine array. The protein concentrations in each supernatant were determined by a BCA^TH^ Protein Assay Kit (Thermo Scientific, Rockford, IL, USA). The cytokine protein levels were corrected for total amount of protein, and the results were expressed as pg/mg or ng/mg.

### 2.8. Foxp3-Positive Cells in the Kidney

We injected BV (1 mg/kg) or same volume PBS once a day for 5 days in Foxp3^EGFP^ mice before cisplatin administration. To visualize and quantify the degree of Treg migration, Zeiss LSM5 confocal microscope (Zeiss, Jena, Germany) was used with kidney samples obtained from Foxp3^EGFP^ mice that were killed at 6 hours after cisplatin injection. And then kidney was sliced into 20 *μ*m cryosection after blood flow. The numbers of Foxp3-positive cell were counted in 10 fields per slide.

### 2.9. Macrophage Infiltration into the Kidney

To evaluate macrophage infiltration into the kidneys after cisplatin administration, immunohistochemical staining for macrophages was performed on paraffin-embedded kidney tissue. The slides were incubated overnight at 4°C with rat anti-mouse F4/80 antibody (dilution 1 : 50; Serotec, Oxford, UK). The primary antibody was localized using the Vectastain ABC Elite Kit (Vector Laboratories) according to the manufacturer's instructions, followed by reaction with a 3,3′-diaminobenzidine substrate-chromogen solution (Vector Laboratories, Burlingame, CA, USA). The slides were counterstained with Harris hematoxylin. The numbers of F4/80-positive cells in each section were calculated by counting the number of positively stained cells in 5 fields per slide at a magnification of ×200.

### 2.10. Tumor-Bearing Mice with a Low Dose of Cisplatin

EL4 lymphoma cells were purchased from KCLB (Seoul, Korea). Cells were cultured in minimum essential medium supplemented with DMEM (Welgene, Daegu, Korea), 10% fetal bovine serum (FBS; GIBCO BRL, Grand Island, USA), and 1% penicillin/streptomycin (Invitrogen, Carlsbad, CA, USA). Cells were grown at 37°C in a humidified atmosphere containing 5% CO_2_. C57BL/6 mice were divided randomly into 3 groups (group I, PBS; group II, PBS + cisplatin; group III, BV + cisplatin). At day 0, EL4 lymphoma cells (2 × 10^6^ cells in 0.1 mL media/mouse) were injected with the same volume of Matrigel (BD Biosciences, San Jose, CA, USA) subcutaneously into the right flank of all mice. BV (1 mg/kg) or PBS was given once a day for 5 days. Thereafter, cisplatin was injected intraperitoneally 3 times (on days 11, 14, and 17) at a concentration of 5 mg/kg each time to groups I and II. The control group received the same volume of PBS. All mice were sacrificed on day 20. The size of the tumor was measured every three days (6 times in total) using calipers. The volume of the tumor was calculated as length × width^2^/2. 

### 2.11. Statistical Analysis

All results are expressed as the means ± SEM. The data were analyzed using two-tailed *t*-test or one-way ANOVA with Tukey's *post hoc* test. Differences were considered significant at *P* < 0.05. 

## 3. Results

### 3.1. Population of Tregs in Spleen

**Figure 1:**
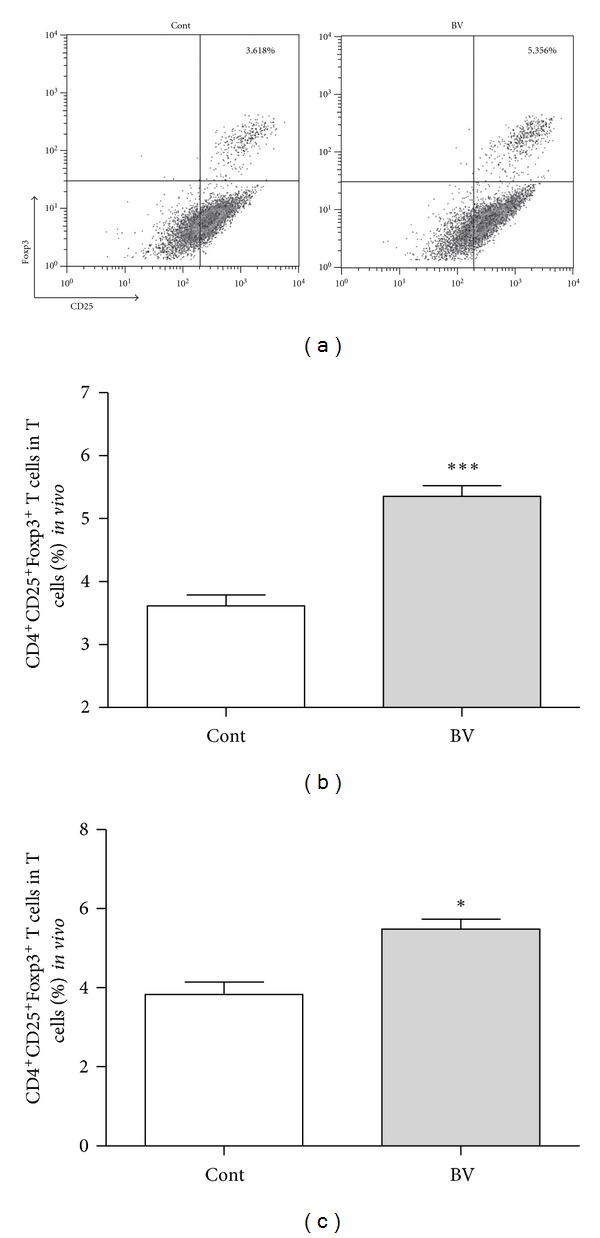
Increased CD4^+^CD25^+^Foxp3^+^ Tregs *in vitro *and* in vivo*. BV (1 *μ*g/mL) was treated in splenocytes separated from Foxp3^EGFP^ mice for 3 days. There was no significant difference in the proportion of dead cells between BV- (20.7%) and PBS-treated (19.3%) groups. This *in vitro* BV treatment significantly increased the number of CD25^+^Foxp3^+^ T cells among the CD4^+^T cells (a, b). And Foxp3^EGFP^ mice were injected with BV (1 mg/kg) or same volume PBS once a day for 5 days. This *in vivo* BV injection also significantly increased the number of CD25^+^Foxp3^+^ T cells among the CD4^+^T cells (c). Tregs were analyzed by flow cytometry gated for CD4-positive cells (a). The values shown indicate the mean ± SEM. ***P < 0.001 versus control; *n* = 5 (b), *P < 0.05 versus control; *n* = 3* in vivo* (c).

To confirm the immune-modulating effect of BV, we isolated splenocytes from sacrificed Foxp3^EGFP^ mice. We treated BV (1 *μ*g/mL) or PBS to incubated cells for 3 days. The CD4^+^CD25^+^Foxp3^+^ cell population was significantly increased in the BV-treated group, compared to the PBS-treated group *in vitro *(cont 3.62 ± 0.17; BV 5.36 ± 0.24, *n* = 5, resp.) (Figures 1(a) and 1(b)). We also examined the population of the CD4^+^CD25^+^Foxp3^+^ cells in spleen *in vivo*. The similar increase in Tregs numbers was observed in BV-injected mice before cisplatin administration (cont 3.84 ± 0.31; BV 5.48 ± 0.25, *n* = 3, resp.) (Figure 1(c)). Three days after cisplatin administration, however, the population of Tregs markedly increased in splenocytes from both of BV- and PBS-injected mice. There was no significant difference in Treg numbers between BV- and PBS-treated animals (cont 13.69 ± 0.52; BV 13.81 ± 0.38, *n* = 4, resp.). 

### 3.2. Survival after Administration of High-Dose Cisplatin

**Figure 2:**
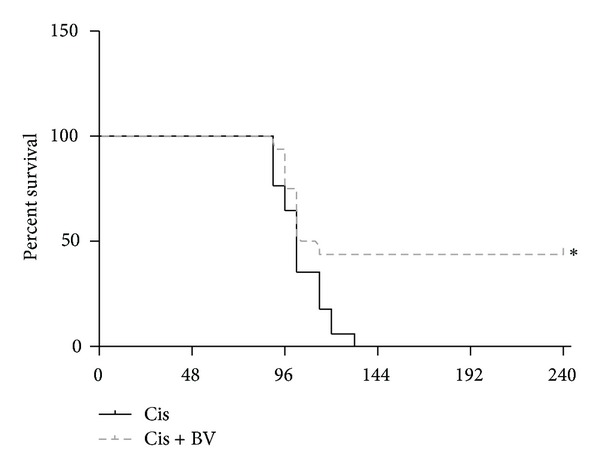
Survival in cisplatin-treated mice. Survival curves of mice treated with 25 mg/kg of cisplatin that were followed up for up to 240 h. BV-treated mice had a 43.75% survival rate, whereas all of the control mice expired. The values shown indicate the mean ± SEM. *P < 0.05 versus control; *n* = 16.

After cisplatin injection (25 mg/kg, i.p.), the mice were monitored for 10 days. All control mice had died by 134 h after cisplatin administration, whereas 7 of the 16 BV-treated mice survived. The BV-treated mice that died at early timepoints expressed features similar to those of the saline-treated mice. However, all of the BV-treated mice that survived to 134 h survived until the end of the experiment (Figure 2).

### 3.3. Protective Effects of BV on Cisplatin-Induced Nephrotoxicity

Mice were treated with intraperitoneal (i.p.) injections of cisplatin (25 mg/kg) after BV treatment. Blood urea nitrogen (BUN) and serum creatinine levels are common indicators of renal function. Blood samples were collected at 24, 48, and 72 h after cisplatin injection, and the levels of blood urea nitrogen (BUN) and creatinine were measured in the 48 and 72 h samples. Creatinine and BUN were significantly increased in the control group compared with BV-treated group (creatinine 48 h: cont 0.16 ± 0.01 mg/dL; Cis 0.83 ± 0.05 mg/dL; Cis + BV 0.46 ± 0.06 mg/dL; creatinine 72 h: cont 0.16 ± 0.01 mg/dL; Cis 3.63 ± 0.14 mg/dL; Cis + BV 2.18 ± 0.34 mg/dL; BUN 48 h: Cont 28.96 ± 1.06 mg/dL; Cis 109.1 ± 14.00 mg/dL; Cis + BV 55.38 ± 5.35 mg/dL; BUN 72 h: cont 22.59 ± 0.81 mg/dL; Cis 267.5 ± 10.73 mg/dL; Cis + BV 193.8 ± 18.12 mg/dL).

**Figure 3:**
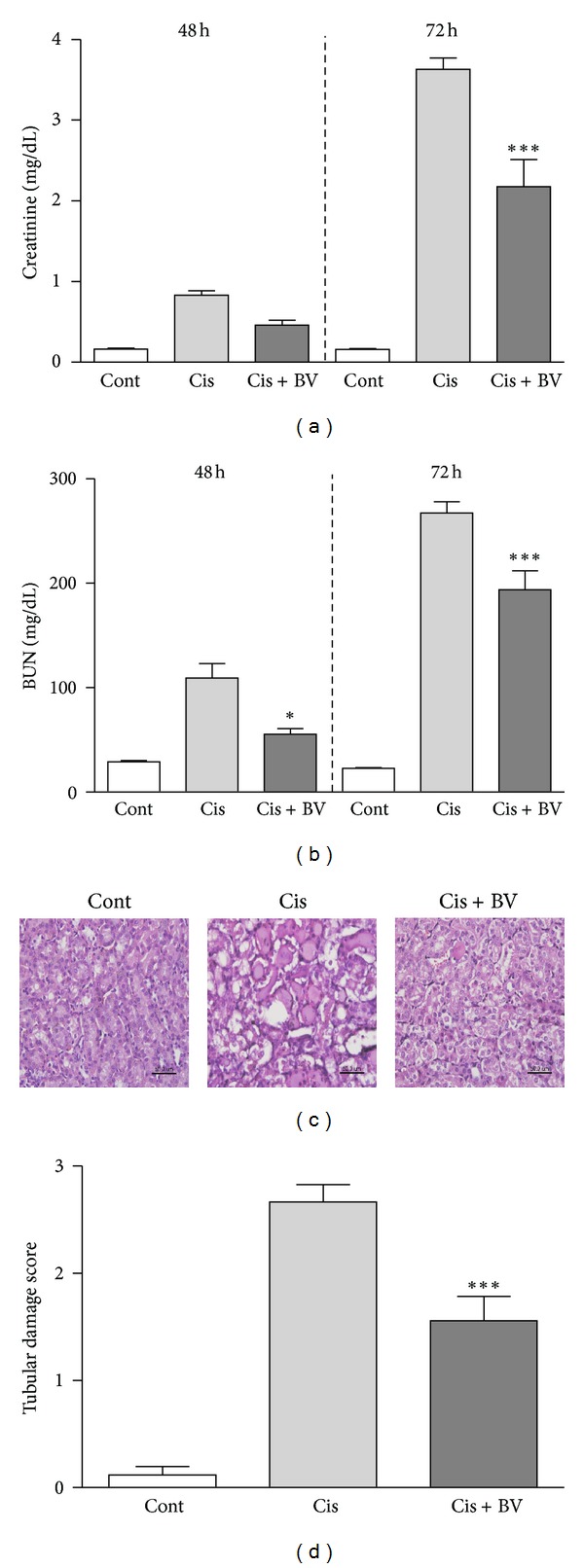
The protective effects of BV on cisplatin-induced nephrotoxicity in mice. Mice received BV (1 mg/kg) once a day for 5 days. The control group received the same volume of PBS. After the fifth administration of BV or PBS, all mice received a single injection of cisplatin (25 mg/kg). Blood samples were obtained at 24 h and 48 h after cisplatin administration, and mice were sacrificed under ether anesthesia at 72 h after cisplatin administration. Blood and kidney samples were extracted for analysis. Blood was obtained at 24, 48, and 72 h after cisplatin injection (*n* = 18). Renal dysfunction was measured as creatinine (a) and BUN (b). Kidney sections were stained with H&E 72 h after cisplatin injection. PBS treatment alone (cont); PBS and cisplatin treatments (Cis); BV and cisplatin (Cis + BV) (400x, bar = 50 um) (c). Tubular injury damage was scored from 0 to 4 (0, none; 1, <10%; 2, 10–25%; 3, 25–75%; 4, >75%) (d). The values shown indicate the mean ± SEM. NS: no significance (*P* > 0.05). *P < 0.05, ***P < 0.001 versus Cis.

To investigate the effects of BV on cisplatin-induced renal damage, we stained kidney tissue sections with hematoxylin and eosin (H&E). Pretreatment with BV significantly reduced the tubular injury score compared to the score in sections from control mice treated with cisplatin alone (renal damage score: Cis 2.667 ± 1.556; Cis + BV 1.556 ± 0.2318) (Figure 3).

### 3.4. Survival in CD25-Depleted Mice

**Figure 4:**
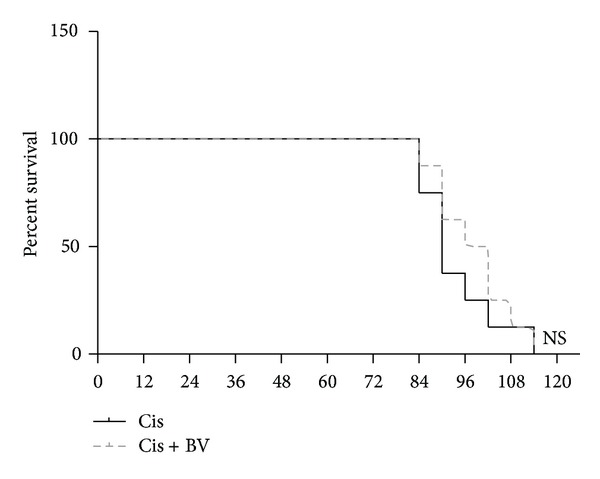
Survival test in CD25-depleted mice. After CD25 depletion, mice were treated with 25 mg of cisplatin/kg intraperitoneally and followed up for up to 114 h to produce survival curves. At 114 h, all of the control mice and BV-treated mice had expired (NS, *P* > 0.05 versus cont; *n* = 8).

After CD25 depletion, all mice received an injection of cisplatin (25 mg/kg, i.p.) and were evaluated for 114 h. All mice in both the control and BV-treated groups had died at 114 h (Figure 4).

### 3.5. The Effects of BV in CD25-Depleted Mice

**Figure 5:**
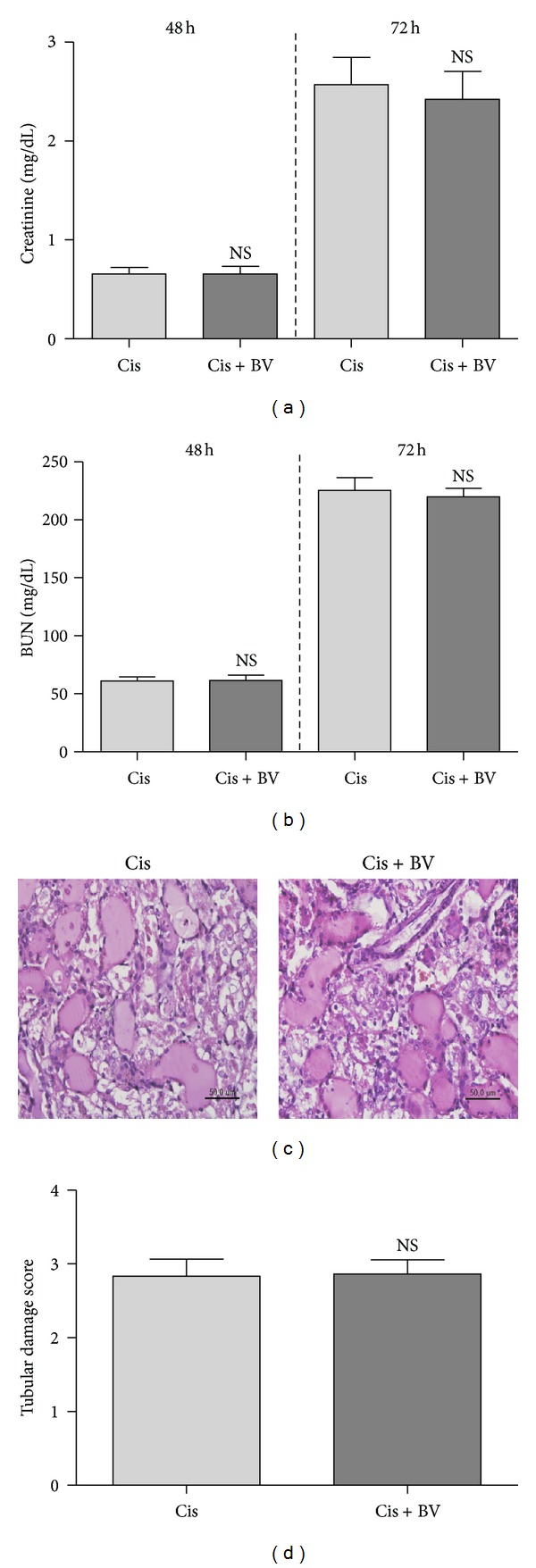
The inefficacy of bee venom in CD25-depleted mice. All mice were treated with anti-CD25 antibody (0.1 mg/mouse, i.p.) twice, before and after BV injection. Nephrotoxicity was induced by cisplatin (25 mg/kg i.p.). Blood was obtained at 24, 48, and 72 h after cisplatin injection (*n* = 12). Renal dysfunction was reflected by the levels of creatinine at 48 h, 72 h (a) and BUN at 48 h, 72 h (b). Kidney sections from CD25-depleted mice were stained with H&E at 72 h after cisplatin injection. PBS treatment alone (cont); PBS and cisplatin treatments (Cis); BV and cisplatin (Cis + BV) (400x, bar = 50 *μ*m) (c). Tubular injury damage was scored from 0–4 (0, none; 1, <10%; 2, 10–25%; 3, 25–75%; 4, >75%) (d). The values shown indicate the mean ± SEM. NS, *P* > 0.05 versus Cis.

To confirm that the effect of BV on cisplatin-induced nephrotoxicity was mediated by Tregs, we tested the combination treatment in a CD4^+^CD25^+^ Treg cell depletion model *in vivo* by treating mice with an anti-CD25 antibody (0.1 mg/mouse, i.p.). We measured the amount of creatinine and BUN in the serum. These results demonstrated that Treg depletion abolished BV-mediated antinephrotoxic effects (creatinine 48 h: Cis 0.66 ± 0.06 mg/dL; Cis + BV 0.66 ± 0.08 mg/dL; creatinine 72 h: Cis 2.57 ± 0.27 mg/dL; Cis + BV 2.42 ± 0.28 mg/dL; BUN 48 h: Cis 61.13 ± 3.58 mg/dL; Cis + BV 61.47 ± 4.73 mg/dL; BUN 72 h: Cis 225.2 ± 11.16 mg/dL; Cis + BV 219.7 ± 7.49 mg/dL). In addition, histological examination revealed that BV had no protective effect on kidney tissues in CD25-depleted mice (renal damage score: Cis 2.83 ± 0.23; Cis + BV 2.87 ± 0.19) (Figure 5).

### 3.6. Proinflammatory Cytokines in Kidney

To evaluate the proinflammatory molecules generated by cisplatin-induced renal injury, the levels of TNF-*α* and IL-6 proteins in the kidney were measured at 72 h after cisplatin administration.

**Figure 6:**
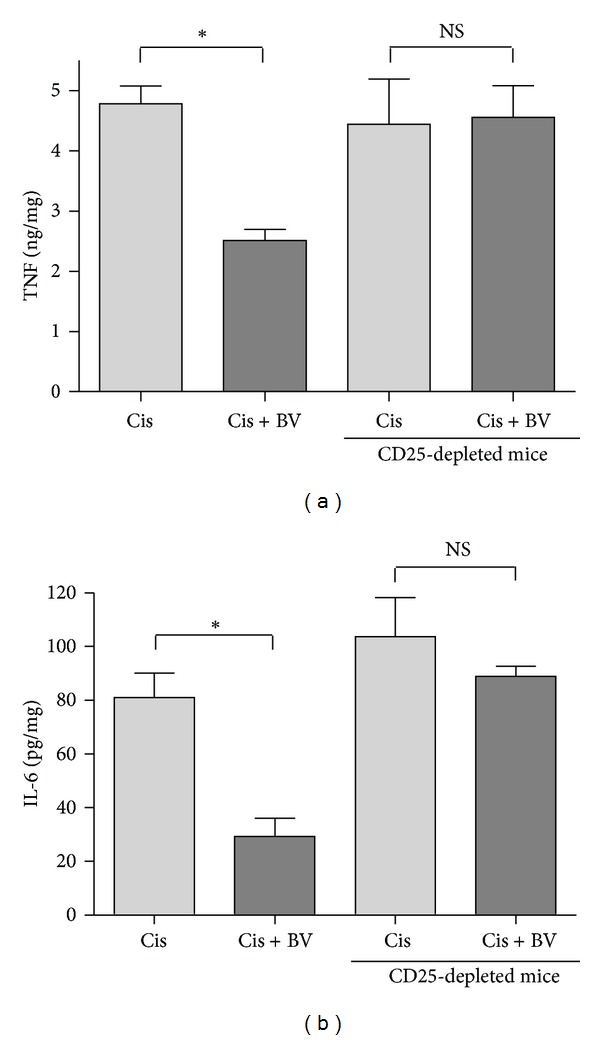
Proinflammatory cytokines in the kidney. Proinflammatory cytokines were measured in kidney tissue from both of normal mice and CD25-depleted mice by ELISA. Renal IL-6 and TNF*α* were decreased in the BV-treated group (Cis + BV) compared with the PBS-treated group (Cis). However, there was no difference between the BV-treated group and PBS-treated group in CD25-depleted mice. The values shown indicate the mean ± SEM. *P < 0.05 versus Cis.

 Only cisplatin-treated mice exhibited increased levels of TNF-*α* and IL-6 at 72 h after cisplatin injection. However, BV-treated mice showed significantly lower levels of these proinflammatory cytokines than did control mice, while Treg depletion caused increases in the levels of these cytokines in both the BV-treated and nontreated groups (Figure 6). 

### 3.7. Migration of Tregs into Kidney

**Figure 7:**
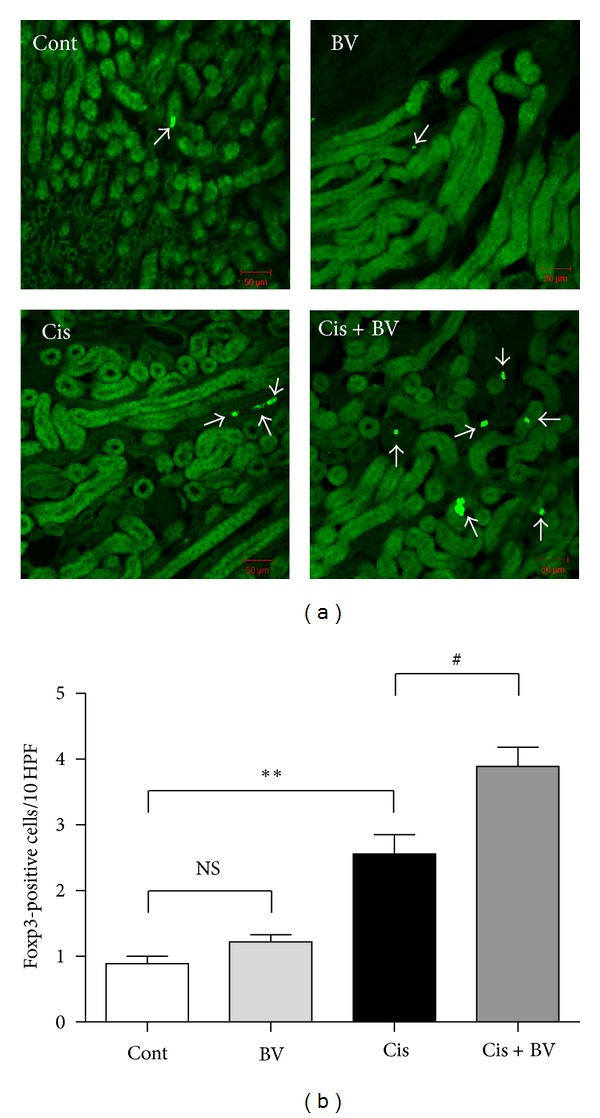
The number of Foxp3-positive cells in the kidney. Tregs in the kidney were detected by EGFP signal under the confocal microscope imaging. Foxp3-positive cells were indicated by arrows (a). There was no difference in the number of Foxp3 positive cells between PBS-treated group and BV-treated group before cisplatin administration. However, Foxp3-positive cells in the kidney increased 6 hours after cisplatin administration. In BV-pretreated group, after cisplatin administration, the number of Foxp3-positive cells in the kidney was significantly higher than in PBS-pretreated group (b). All data are presented as the mean ± SEM. NS, *P* > 0.05, *P < 0.05 versus cont, and ^#^ *P* < 0.05 versus Cis, as determined by one-way ANOVA followed by Tukey's multiple comparison test.

Confocal microscopy was used with kidney samples obtained from Foxp3^EGFP^ mice that were killed at 6 hours after cisplatin injection. More Foxp3-positive cells were observed in the kidney in BV-pretreated group, compared to the PBS-treated group, 6 hours after cisplatin injection (Figure 7), suggesting the increased migration of Tregs into the kidney in the early phase of nephrotoxic process caused by cisplatin.

### 3.8. Effects of BV on Cisplatin-Induced Macrophage Infiltration

**Figure 8:**
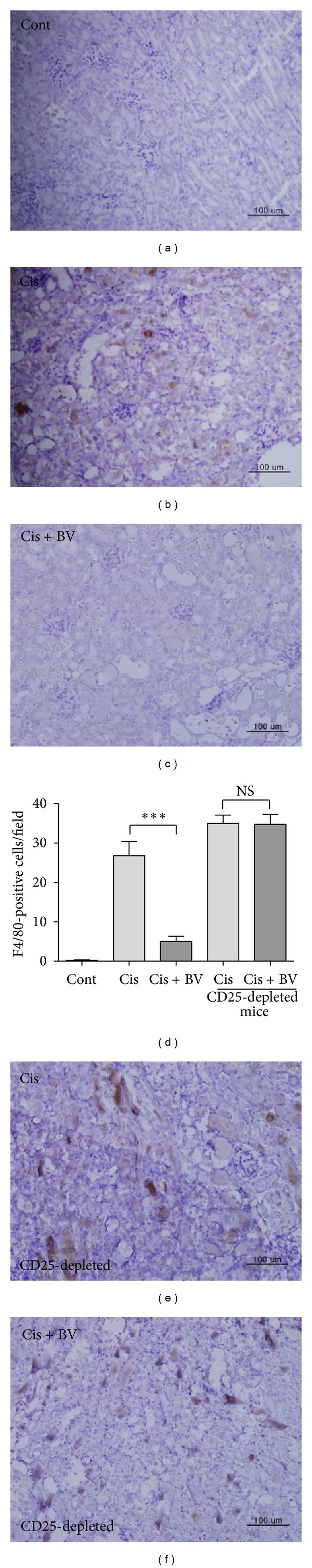
Macrophage infiltration into kidney. Macrophage accumulation in the kidney was detected by immunostaining for F/480-positive cells at 72 h after cisplatin administration. The number of F4/80-positive cells in each section was counted in 10 fields per slide at an original magnification of 200x. The infiltration in BV-treated mice was compared with those of PBS-treated mice and CD25-depleted mice. The values shown indicate the mean ± SEM. ***P < 0.001 versus Cis.

Macrophages were counted in the kidney sections at 72 h after cisplatin administration. F4/80-positive macrophages were rarely detected in BV-treated mice compared with control mice, whereas more infiltration of macrophages was measured in CD25-depleted mice (Figure 8). 

### 3.9. The Influence of BV on Cisplatin-Induced Anticancer Effects

To determine whether the preadministration of BV influenced the anticancer effects of cisplatin, the experiment was repeated in a tumor-bearing (EL4 lymphoma) mouse model with a low dose of cisplatin (5 mg/kg). Cisplatin was injected 3 times at a concentration of 5 mg/kg. The mice were weighed and the size of the tumor was measured over the whole experimental period. All mice were sacrificed on day 21, and the separated tumors were collected. 

**Figure 9:**
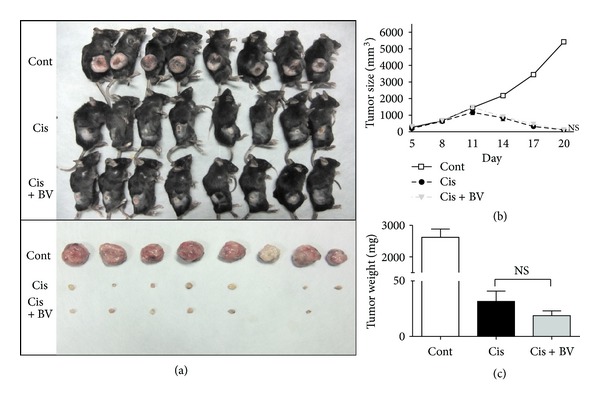
Influence of BV on the antitumor effect of cisplatin. All mice received injections of EL4 lymphoma cells (2 × 10^6^/0.1 mL) s.c. into the right flank. BV (1 mg/kg) was injected once per day for 5 days from day 5 of tumor inoculation (*n* = 8). Cisplatin was injected 3 times (on days 11, 14, and 17) at a concentration of 5 mg/kg. All mice were sacrificed at day 20, and the tumors were photographed (a). The tumor size was measured every three days after BV injection for 16 days. After sacrifice, the tumors were fully separated from the mice, and the size (b) and weight (c) of the tumors were measured. All data are presented as the mean ± SEM. ***P < 0.001 versus cont; NS, *P* > 0.05 versus Cis, as determined by one-way ANOVA followed by Tukey's test.

 Tumor size in all groups of mice was gradually increased before cisplatin administration (until day 11 in Figure 9(b)). There was no significant difference in tumor size before cisplatin administration between BV and PBS groups. In addition, irrespective of BV pretreatment, repeated administrations of cisplatin exerted very potent anticancer effects (a remarkable reduction in tumor size) in EL4 tumor model, resulting in no difference in tumor size between PBS- and BV-preinjected groups (Figures 9(a)–9(c)). In contrast, tumor size in control mice (tumor model without cisplatin) continuously increased after cisplatin administrations (Figure 9(b)). The amounts of creatinine and BUN were within the normal range in all groups (data not shown). These results suggest that BV has not a big influence on the anticancer effects of cisplatin.

## 4. Discussion

The development of side effects after cisplatin treatment is common, and acute renal failure may develop after exposure to a single dose [2]. Acute renal failure, as induced by cisplatin, leads to the generation of danger signals by tubular cells, resulting in inflammation mediated by T lymphocytes and NK cells [15]. Tregs are lymphocytes with immunosuppressive properties and play a pivotal role in the maintenance of immune tolerance [5]. Previous studies have reported that the anti-inflammatory actions of Tregs can protect against renal injury [16–18]. Our recent study demonstrated that CD4^+^CD25^+^ Tregs attenuate cisplatin-induced nephrotoxicity *in vivo *[8]. Thus, we evaluated the effects of many candidate agents on CD4^+^CD25^+^Foxp3^+^ Tregs *in vitro* and found that BV potently increases the population of CD4^+^CD25^+^Foxp3^+^ Tregs. Moreover, it was shown that BV has remarkable renoprotective effects in NZB/W lupus mice [12]. Based on these lines of evidence, we hypothesized that BV may have therapeutic effects on cisplatin-induced nephrotoxicity through the upregulation of Tregs.

In this study, we first showed that BV indeed increases the population of CD4^+^CD25^+^Foxp3^+^ Tregs in isolated mouse splenocytes *in vitro* and *in vivo* before cisplatin administration (Figure 1), corroborating the results of our previous study [12]. Three days after cisplatin administration, however, the population of Tregs markedly increased in splenocytes from both of BV- and PBS-injected mice with no significant difference in Treg numbers between the two groups. We further examined how many Tregs are migrated into the kidney after cisplatin administration. We found that more Tregs were migrated into the kidney 6 hours after cisplatin injection in BV-pretreated mice than in PBS-pretreated mice (Figure 7). Next, we found that BV has protective effects on cisplatin-induced renal dysfunction and injury. Serum BUN and creatinine are typical indicators of renal function, and an increase in these values could indicate renal failure [19]. Our data showed that cisplatin treatment significantly increased the BUN and serum creatinine levels in the saline-treated control group and that this increase was prevented by BV treatment. The histological analysis of the degree of renal tubular injury (epithelial necrosis) also suggests that BV can effectively prevent the structural damage of renal tissues. The degree of macrophage infiltration into the kidney is a major parameter used to predict the progression of renal disease, and activated macrophages express several inflammatory mediators [20], including TNF-*α* and IL-6, which play important roles in the pathogenesis of cisplatin-induced renal injury [21, 22]. Our data showed that BV treatment in cisplatin-injected mice decreased the expression of proinflammatory cytokines as well as macrophage infiltration in the kidney. Because renal toxicity is the main cause of death in cisplatin-injected mice [23], we evaluated the survival of BV- or saline-treated mice after cisplatin administration. The BV-treated mice showed a higher survival rate than did the saline-treated control mice. Collectively, these results might suggest that Tregs in spleen increase in number after BV treatment before cisplatin administration, which cause more migration of Tregs into the kidney in the early phase of cisplatin-induced nephrotoxicity. Such action might lead to the protective effects of BV on cisplatin-induced nephrotoxic events as shown in the present study. This is supported by our previous study [8] showing that adoptive transfer of Tregs to mice (i.v.) caused increased migration of Tregs into the kidney 6 h after cisplatin administration and then attenuated the renal injury. The renoprotective effects of BV in cisplatin-injected mice in the present data are comparable to the results shown in Treg-transfused mice [8]. 

Since BV is known to have direct anti-inflammatory and antitumor effects [24, 25], we cannot rule out the possible involvement of Tregs-independent mechanisms of BV in the present results. However, we demonstrated that depletion of Tregs using anti-CD25 antibody injection completely inhibited the BV effects on cisplatin-induced nephrotoxic events. Therefore, our findings strongly suggest that BV could prevent cisplatin-induced severe side effects in the kidney, at least partly, by modulating the Tregs.
